# Perceptions and experiences of lifestyle interventions in women with polycystic ovary syndrome (PCOS), as a management strategy for symptoms of PCOS

**DOI:** 10.1186/s12905-021-01252-1

**Published:** 2021-03-17

**Authors:** Susan Arentz, Caroline A. Smith, Jason Abbott, Alan Bensoussan

**Affiliations:** 1 NICM Health Research Institute, Western Sydney University, Locked Bag 1797, Penrith, NSW 2751 Australia; 2 School of Women’s and Children’s Health, University of New South Wales, Barker Street, Randwick, NSW 2031 Australia

**Keywords:** Polycystic ovary syndrome, Healthy lifestyle, Exercise, Women’s health

## Abstract

**Background:**

The international clinical practice guidelines for PCOS emphasize diet and exercise as first-line management of clinical signs and symptoms. This study aimed to describe the patterns, perceptions and experiences of lifestyle interventions for women in the community with PCOS.

**Method:**

An electronic survey of 493 members of two PCOS consumer support groups, collected by cloud-based Survey Monkey, described women’s types and patterns of diet and exercise, experiences and perceptions of effectiveness. Women were recruited from the Polycystic Ovary Association of Australia (POSAA) and from the Facebook group, PCOS University Research Group. Associations between participants perceptions of effectiveness, and diet types and exercise patterns were assessed using logistic regression. Response bias for the POSAA group was assessed with a continuum of resistance model.

**Results:**

91% of POSAA members and 311 Facebook group members aged 16–50 years responded to the survey. Nearly all women reported adjusting their dietary and exercise practices with the aim to improve their health and/or PCOS (82% and 73% respectively), however less than 13% reported achievement of health goals (12.2% and 8.1% respectively). Low carbohydrate, high protein diets, and vigorous activity were associated with self-perceived effectiveness (r.0.16, p < 0.01; r.0.15 p < 0.01 and r.0.2 p < 0.01 respectively). Barriers for lifestyle interventions included psychosocial factors. Response bias was not assessed for the Facebook group, however self-reported PCOS aligned with prevalence of clinical phenotypes and suggests results are generalizable to clinical populations of women with PCOS, who are responsible for self-directing and administering lifestyle interventions to manage their PCOS.

**Conclusions:**

Perceptions of effectiveness for lifestyle interventions by women with PCOS may be complicated by a lack of rigorous evidence. The strength of recommendations in clinical practice guidelines may be enhanced by clinical trials investigating flexible and feasible lifestyle interventions for women in the community with PCOS.

**Supplementary Information:**

The online version contains supplementary material available at 10.1186/s12905-021-01252-1.

## Background

Polycystic ovary syndrome (PCOS) is the most common reproductive endocrinopathy affecting up to one in five women of reproductive age [1, 2]. The cause of PCOS remains unknown however increased prevalence coincides with a global increased incidence of metabolic syndrome. Metabolic disturbance is associated with increased adiposity and clinically worsens menstrual cyclicity, hyperandrogenism, ovulation rates, fertility, maternal and neonatal outcomes and increases women’s risks for diabetes, cardiovascular disease and cancer [2, 3].

The international evidence based guidelines (EBG) for the management of PCOS highlights personal lifestyle behaviours to reduce and prevent risk factors significantly associated with increased body weight in women with PCOS [4]. Diet and exercise interventions aimed at weight reduction and prevention of weight gain are first-line management strategies due to their significant impact on clinical outcomes, including reproductive endocrinology and clinical signs and symptoms [3–5]. However, current evidence for lifestyle intervention for women with PCOS is not well established. Many studies informing the evidence base are underpowered and report high risk of bias, particularly from high attrition [5–8]. The effectiveness of specific dietary and exercise practices for women centred outcomes has not been established. It has not been shown that if, and to what degree diet and exercise performed outside the confines of study intervention environments correlate with improved health status for women with PCOS in the community [9].

Dietary and exercise practices of women may be influenced by a wide range of ecological factors including inter and intrapersonal, environmental and psychosocial characteristics of individuals plus levels of health literacy and potential for self-efficacy [10–14]. Health promoting behaviours by women with PCOS may be complicated by a lack of evidence informing women of the benefits of lifestyle intervention [4], and may exacerbate psychological morbidity, which has a high prevalence in women with PCOS [15], and frustration with current medical treatments [16, 17]. To date there are few data describing the views of women with PCOS for engagement and optimal effectiveness of dietary and exercise strategies in community-based environments.

This study has two aims; to describe the experiences of women with PCOS living in the community, and to explore their perceptions about the effectiveness of dietary and exercise interventions for PCOS.

## Method

The study was approved by the University of Western Sydney Human Research and Ethics Committee H9341. An anonymous electronic survey was undertaken to describe the experiences of community-based women with self-reported PCOS, and associated symptoms, and the role of diet and exercise and other interventions which may contribute to the management of PCOS symptoms and wellbeing. Women with PCOS seeking health support as members of two consumer support networks, the land based Polycystic Ovary Syndrome Association of Australia (POSAA) and the digital social network site (SNS), Facebook, were approached and invited to participate. Participants self-selected from POSAA following direct email to 258 financial members between November 2011 and September 2013. A second sample from Facebook was generated by group members response to an invitation to participate in the survey and a link placed on the ‘wall’ of the Facebook group page www.uws.edu.au/pcosfacebook. Wall postings occurred bi-monthly between February 2013 and May 2013. Included were women who self-identified as having PCOS, with access to an electronic device and with English language skills. All participants were members of PCOS support networks and self-reported a prior diagnosis of PCOS.

### Questionnaire design

A 37-item questionnaire was designed to describe the signs and symptoms of women with PCOS and women’s use of medical treatment and diet and exercise to manage PCOS. (Additional file 1). Fifteen items sought information regarding dietary and exercise practices and included types of diets and exercise, types and patterns of exercise practice in terms of frequency, duration and intensity. Moderate intensity exercise was described as causing an increased breath and heart rate but still able to talk. Vigorous exercise was described as causing markedly increased breath and heart rate and not being able to talk during activity. Additional questions examined barriers to engagement including reasons for not partaking or disengaging with therapeutic diet and exercise practices and questions about self-perceived achievement of goals and satisfaction with diets and exercise. Socio-demographic characteristics were also collected. Multiple response options were available for participants to indicate which signs and symptoms of PCOS they were experiencing, and the types of dietary and exercise practices used. Item three sought frequency of ten signs and symptoms typically associated with PCOS including ‘feeling depressed due to PCOS,’ with an open-ended option for others. Frequency was reported on a six-point Likert scale ranging from ‘all the time’ to ‘not at all.’ A cover letter informed participants’ that consent was implied following electronic submission, and that responses could not be withdrawn due to anonymity of the survey. The questionnaire took 15–20 min to complete. The stability of the questionnaire was tested on a pilot sample of eight women including four with PCOS to review the applicability and accuracy of the questionnaire.

The electronic survey was loaded onto Survey Monkey [18] and made available to both members of POSAA and members of the Facebook group. A single response function was applied to each member and to devices.

### Data analyses

Responses were analysed using the statistical package SPSS version 21.0 [19]. Categorical responses were reported as percentages and proportions with 95% confidence intervals. Relationships between variables were explored using Pearson’s correlation (two tail) for parametric data and Spearman’s Rho correlations or Gamma co-efficient (collapsed less than six variables) for non-parametric measurements. Associations between perceptions of effectiveness (dependent variable) and type and pattern of diet and exercise (covariates) were assessed using logistic regression. A p value of 0.05 was considered statistically significant.

Non-response bias was assessed using a continuum of resistance model in which late responders were viewed as proxy non-responders [20]. Participants who responded to the survey four months or more after the final reminder, were classified as late responders. This method was chosen due to the likelihood of early response by women who found the topic interesting [21]. The impact of non-response bias was assessed by comparing early and late responders.

## Results

Four hundred and ninety-three participants aged between 16–50 years responded to the survey; 235 of the 258 invited members of the POSAA group responded (91.1%) including 53 late responders, and 311 women replied to snowballing using the University Research Facebook group page. No differences in demographics or PCOS signs and symptoms were found between early and late responders or the two consumer support groups (POSAA and Facebook) (Additional file 2: Tables S1 and S2).

Four hundred and thirty-four (88.0%) subjects completed all parts of the survey, with the remainder using the skip patterns inserted into the electronic survey or completing only part of the survey.

The demographics of participants are presented in Table 1. Most women were aged between 25 and 34 years (n = 237, 48.1%, ± 4.41, 95%CI), were tertiary educated (n = 323, 76.2%, ± 4.05), and employed, (335, 67.9%, ± 4.12).

**Table 1:** Demographic characteristics of Survey participants

	Frequency N = 493	Valid %	95% Confidence Interval
*Age*			
16–17	3	.7	± 0.79
18–24	72	17.0	± 3.58
25–29	126	29.7	± 4.35
30–34	111	26.2	± 4.19
35–40	71	16.7	± 3.55
41–44	25	5.9	± 2.24
45–50	16	3.8	± 1.82
Missing data	69	14	
*Education*			
Complete high School			
Yes	371	87.5	± 3.15
No	48	11.3	± 3.01
Currently enrolled	5	1.2	± 1.04
Missing data	69	14	
Completed tertiary education			
Yes	323	76.2	± 4.05
No	55	13.0	± 3.0
Currently enrolled at a tertiary inst	46	10.8	± 2.95
Missing data	69	14	
Total	493		
Qualification from TAFE	103	20.9	± 3.59
Private institution Diploma	70	14.2	± 3.08
University degree	221	44.8	± 4.39
Missing data	69	14	
*Ethnicity- country of birth*			
Australia	302	61.3	± 4.3
Other	121	24.5	± 2.08
Missing data	70	14.2	
*Employment*			
Home duties	117	23.7	± 3.75
Self-employed	38	7.7	± 2.35
Student	80	16.2	± 3.25
Employed part time	103	20.9	± 3.59
Employed full-time	194	39.4	± 4.31
Other	12		± 1.35
*Private Health Insurance*			
Yes	275	65	± 4.55
No	148	35	± 4.55
**Missing data**	**70**	**14.2**	

Over 75% of participant’s reported being over-weight, (n = 357, 77.3% ± 3.82). Two thirds of responders reported experiencing a late menstrual period (n = 304, 67.1%, ± 4.33, 95% CI) and hirsutism (n = 327, 68.8%, ± 4.17). Over half reported infertility (n = 248, 60.2%, ± 4.73) and insulin imbalances (n = 251, 55.7% ± 4.58), and nearly half reported feeling depressed as a result of having PCOS (n = 200, 44% ± 4.56). Women reporting a late menstrual period (oligo/amenorrhea) were significantly more likely to report, being very overweight (r.0.29, p < 0.01) and infertility (r.0.45, p < 0.001) but unlikely to report feeling depressed due to PCOS (r.0.08, p = 0.10). Women reporting hirsutism were more likely to report depression (r.0.24, p < 0.01) (Table 2).

**Table 2:** Self-reported correlation of PCOS signs and symptoms

Signs and symptoms	Depression	Waist more cm’s than hip	Infertility	Cramps
*Late period*				
Pearson Correlation	.079	.273**	.450**	.193**
Sig. (2-tailed)	.097	< .01	< 0.01	< 0.01
N	443	440	419	398
*Hirsutism*				
Pearson Correlation	.241**	.301**	.082	.003
Sig. (2-tailed)	< 0.01	< 0.01	.092	.957
N	453	448	426	400
*Overweight*				
Pearson Correlation	− .013	.222**	.225**	.079
Sig. (2-tailed)	.781	< 0.01	< 0.01	.112
N	458	455	433	410
*Very overweight*				
Pearson Correlation	.019	.228**	.408**	.237**
Sig. (2-tailed)	.690	< 0.01	< 0.01	< 0.01
N	443	440	422	395
*Blood sugar/insulin imbalance*				
Pearson Correlation	.112*	.225**	.691**	.222**
Sig. (2-tailed)	.018	< 0.01	< 0.01	< 0.01
N	449	448	426	402

**Correlation was significant at the 0.01 level (2-tailed)

*Correlation was significant at the 0.05 level (2-tailed)

cm’s: centimetres

Sig: significance

N: frequency of valid cases

### Management of PCOS

The respondents used various medical therapies to manage PCOS (Table 3). Over sixty five percent (± 4.21) had used the oral contraceptive pill, 62.7% (± 4.26) had used other pharmaceuticals including ovulation induction, hypoglycaemic and anti-androgen drugs.

**Table 3:** Medical interventions used by participants

	Frequency	%	Missing	N
Oral contraceptive pill	322	65.3	171	493
Pharmaceuticals: ovulation induction, anti-androgens hypoglycaemics etc	309	62.7	184	493
Ovarian stimulation as part of an IVF cycle	58	11.8	435	493
Surgery	49	9.9	444	493
No medical treatment	10	2	483	493

### Lifestyle interventions

#### Diet

Over 82% (± 3.36, n = 406) of respondents reported altering their dietary intake during the previous five years for health reasons. Over 57% (± 4.37, n = 282) reported consuming a diet specifically to manage symptoms associated with PCOS and had tried various types of diets. The most frequently used was the low glycaemic (GI) index diet (50.1%, ± 4.41, n = 247), followed by the low calorie diet (36.9%, ± 4.26, n = 181), low carbohydrate diet (33.7%, ± 4.17, n = 166) and low fat diet (32.9%, ± 4.15, n = 162).

Women were asked to indicate their reason for dieting. Most respondents (72.7%, ± 3.93) modified their diet with the aim to lose weight. Other reasons included maintaining general health, 59% (± 4.64), to manage blood sugar levels 37.3% (± 4.56), to improve body composition 15% (± 3.37) and for social or religious reasons 7.4% (± 2.47).

The majority of women reported they did not fully achieve their health goals using dietary changes. Over thirty three percent (± 4.5 n = 140) reported they did not achieve their weight loss goals or any positive health changes from dietary practices. Forty six percent (± 4.78, n = 195) reported that dietary practices had contributed partly to positive health changes and a small number of women (n = 51) reported fully achieving their health goals using dietary practices (12.2%, ± 3.13).

Positive associations between types of diets and achievement of health goals were found between the low carbohydrate diet (r. 0.16, p < 0.01, N = 419) and the high protein diet (r. 0.15, p < 0.01, N = 419).

#### Exercise

Most women, (73.1%, ± 4.2, n = 313) reported regular participation in moderate or vigorous exercise. Women were engaged in a variety of different forms of exercise, most exercisers reported practicing two or more types (97%, ± 4.57, n = 305). Over half of participants (61.3%, ± 3.13) were engaged in formal exercise including supervision provided by a personal trainer, participation in a team sport or attending a gymnasium and lifting weights, running, swimming, cycling or supervised classes. Over one third (37.5%, ± 4.27) of women reported incidental activity as exercise. Other exercise included Pilates, dance, personal trainer, tennis and team sports.

Most respondents reported multiple reasons for exercising, with 80% (± 4.45) using exercise to feel better in themselves, 75% (± 4.79) to induce weight loss and 60% (± 5.45) to prevent weight gain. Sixty percent of responders (± 5.45) were exercising specifically to manage their PCOS.

One hundred and fifteen participants did not exercise (26.9%, ± 4.2). Reasons for not exercising included lack of time (n = 109), felt embarrassed (n = 93), and financial costs (n = 52). Fifty-one cited other reasons such as feeing unmotivated, depressed, and co-morbid overweight conditions including back pain and arthritis.

Most women reported participation in both moderate and vigorous exercise. Over 88% (± 3.5, n = 277) of exercising women, regularly engaged in moderate exercise, and nearly half (48.7% ± 6.04, n = 128) in vigorous exercise. Seven women reported not doing any moderate exercise at least once per month, and 88 women (28.3%, ± 4.67) were not engaged in any vigorous exercise at least once per month and 87 women, no vigorous exercise at all.

Most women exercised two to four times per week (42.0%, ± 5.46, n = 132). Most responders exercised moderately for 15–30 min per session (46.1%, ± 5.49, n = 146,) and just under 40% exercised vigorously for 30–60 min (39.9%, ± 5.44, n = 134).

Nearly forty percent (39.4%, ± 5.35, n = 127) reported non achievement of health goals with exercise. The majority reported part achievement using exercise (51.8%, ± 5.46, n = 166), and only eight percent (± 2.93, n = 25) of respondents felt they had achieved their health goals with exercise.

We found that increased frequency and duration of exercise was significantly associated with the achievement of health goals and an inverse association between the duration of moderate exercise and the achievement of health goals. (Table 4).

**Table 4:** Duration of exercise and self-perceptions of the achievement of health goals

		Vigorous exercise duration	Moderate exercise less than 60 min	Moderate exercise more than 60 min
Self-perceived achievement of goals	Pearson Correlation	.196**	.247**	− .006
	Sig. (2-tailed)	.000	.000	.919
	N	320	320	320

**Correlation was significant at the 0.01 level (2-tailed)

*Correlation was significant at the 0.05 level (2-tailed)

Sig: significance

N: frequency of valid cases

## Discussion

This study highlights the views of women in the community with PCOS and describes their self-reported dietary and exercise lifestyle behaviours and perceptions of effectiveness. Nearly all women reported adjusting their dietary and exercise practices with the aim to improve their health and/or PCOS, however less than 13% reported achievement of health goals. Participants reported a range of PCOS symptoms including being overweight, reproductive disorders and negative emotional and mental health and frequent engagement with positive health behaviours as part of self-care management, with the primary aims to lose weight and manage PCOS.

Women with PCOS in the community have been found to lack support and information for self-managing PCOS symptoms using lifestyle interventions [9, 17]. In a survey of 1385 women with PCOS, only 11.9% reported satisfaction with information provided about beneficial diet and exercise [17]. In the same study, less than 4% of participants were satisfied with information provided about the emotional features of PCOS, with no information or support being offered in most cases [17]. Lack of information and support for community-based women with PCOS may have influenced the low perceptions of efficacy for lifestyle interventions found in the present study. Women’s information needs include details about types and patterns of safe and effective lifestyle interventions, and mediators for success and goal achievement including the impact of social support and influence of expectations and experiences of lifestyle interventions [15, 22].

Views of community-based women with PCOS presented here contrast with findings of a randomized control trial (RCT) evaluating diet and exercise on the motivators and barriers of exercise for women with PCOS [8]. This clinical investigation found women’s perceptions of exercise were significantly improved over 20 weeks in all three study arms (diet only, diet plus aerobic exercise and diet plus aerobic and progressive resistance exercises), and correlated with improved anthropometry, health related quality of life and less depression [8]. Greatest perceived benefits were on the psychological outlook and social interaction subscales of the Exercise Benefits/Barriers Scale (EBBS) (p ≤ 0.001). Other significant findings were reduced barriers on subscales of exercise milieu (atmosphere), time expenditure and physical exertion (p ≤ 0.003). The contrasting findings found in the present study may be explained by the recruitment strategy of women with PCOS from different populations and by differences in the nature and structure of lifestyle interventions.

The present study recruited participants from consumer support groups in order to assess the views of women in the community, whereas recruitment to the clinical investigation was conducted via public advertisements via medical speciality clinics. Women with PCOS seeking medical support have been shown to display different characteristics of PCOS compared to community-based women [2] which may explain variable perceptions of exercise interventions found in the present study. In addition, the nature of the interventions provided in the clinical trial were structured with weekly contacts, compared to self-informed, self-initiated, self-administered and self-accounted lifestyle interventions typically used by women in the community. Although nearly two thirds of participants reported engagement with supervised/structured exercise, a lack of specific information about evidence-based lifestyle recommendations for PCOS, may have limited guidance of exercise supervisors, with respect to the types and patterns of exercise provided, and a lack of information may have mediated women’s expectations and experiences of efficacy [23].

It is unclear which dietary and exercise strategies are optimal for women with PCOS. Current recommendations based on reduced caloric intake, combined with moderate energy expenditure, are described as being most likely to produce sustained weight loss and favourable endocrine and reproductive outcomes [5, 24–27]. Women in this study reported similar optimal dietary and exercise practices on symptoms of PCOS, with greatest effectiveness reported for low carbohydrate and high protein diets compared to low calorie diets. Women’s perceptions of effective exercise practices highlighted the importance of including vigorous activity, however many respondents reported no engagement in any vigorous activity at all. Various reasons were cited, including time limitations, feelings of embarrassment and personal injury. Additionally, exercise preferences of women with PCOS may favour non-vigorous activity. A study into the comparative exercise practices of women with and without PCOS, controlled for BMI, (n = 163) showed that women with PCOS were less likely to engage in vigorous activity [28] and was strongly determined by personal circumstances and characteristics.

Despite the present study finding most women’s exercise activity aligned with recommendations in clinical practice guidelines [29], the respondents perceptions of low efficacy elucidates a gap in information [17] and a need for guidance despite the absence of high quality evidence [5]. Women in the community still need to make decisions about patterns and types of lifestyle to manage PCOS. Their perceived low efficacy may be complicated by the absence of rigorous information and reflect the trial and error process of self-directed and self-funded searches for the most effective and feasible lifestyle interventions for their case and circumstances.

This study contributes to our understanding of women with PCOS living in the community, and their experiences using lifestyle interventions to manage symptoms and health. This research gives voice to community-based women with PCOS and identifies important modifying factors for consideration in future clinical practice recommendations [30]. It may also guide pragmatic clinical trial investigations of flexible lifestyle interventions, conducted in community settings with real-world characteristics [31] in order to improve the quality of evidence and strengthen recommendations of clinical practice guidelines [4].

### Limitations and strengths

There are some methodological limitations of this study including the non-response and selection bias of recruitment from Facebook support groups. We were not able to assess unsolicited invitations from group administrators and response bias analyses was not possible. Women volunteered to participate, which may have introduced response bias with responders potentially reporting more positive or negative experiences of PCOS and perceptions of lifestyle effectiveness compared to non-responders [32]. Generalizations are limited to computer literate women with English language skills, as members of support networks and other groups of women with PCOS may be underrepresented. The questionnaire was not validated against medical records for PCOS lifestyle interventions; however, it was designed to investigate women with PCOS in the community and their perceptions, usage and experiences of lifestyle interventions, which may not coincide with the views of clinical groups. PCOS and physical characteristics including BMI and anthropometric measurements were self-reported and at risk of inaccuracy, however a similar symptom profile to clinical populations [1, 15, 33] was described, and these results may be generalisable to women with medically diagnosed PCOS, living in the community. Genetic analyses of PCOS susceptibility has demonstrated consistency between woman’s self-reports of the condition and rigorous diagnoses [34, 35].

## Conclusion

Women in the community with PCOS report frequent engagement in a variety of dietary and exercise practices according to the clinical practice guidelines, and they perceive their health goals are partially met. Self-perceived lack of efficacy for dietary and exercise interventions may be due to the lack of high-quality evidence of effectiveness for specific lifestyle interventions. The quality of evidence might be improved by pragmatic RCTs of community-based samples, comparing lifestyle interventions on outcomes of value to women with PCOS.

## Supplementary Information

**Additional file 1: **Survey instrument.

**Additional file 2: Table 1:** Comparison of early and and late responders and **Table 2:** Characteristics of POSAA and Facebook samples.

## Data Availability

The datasets and materials used and analysed during the current study are available from the corresponding author on reasonable request.

## Abbreviations

BMI: Body Mass Index
EBBS: Exercise Benefits/Barriers Scale
EBG: Evidence Based Guidelines
GI: Glycaemic Index
PCOS: Polycystic Ovary Syndrome
POSAA: Polycystic Ovary Association of Australia
RCT: Randomized Control Trial
SNS: Social Network Site
