# Supplementary material for: Perceptions and experiences of lifestyle interventions in women with polycystic ovary syndrome (PCOS), as a management strategy for symptoms of PCOS
Source: BMC Womens Health. 2021 Mar 17;21:107. doi: 10.1186/s12905-021-01252-1 (PMC7968330; doi:10.1186/s12905-021-01252-1)
Supplement: Supplementary file 1 — Additional file 1: Survey instrument. [file 12905_2021_1252_MOESM1_ESM.docx]

A survey to explore the use of Complementary Medicine and self help measures to assist with symptom management of polycystic ovary syndrome.

This survey is part of a study is being carried out by the University of Western Sydney with the support of the Polycystic Ovary Association of Australia (POSAA) to provide a clearer understanding of the use of self prescribed therapies and complementary medicine or therapies by women with polycystic ovary syndrome (PCOS). Your answers will help to better understand the experiences of women with PCOS and for health providers to provide better care. The main therapies in this questionnaire include diet, exercise and physical activity and complementary medicine and therapies. Many complementary medicines do not have a clear evidence base for their effectiveness and this study does not in any way imply that POSAA endorse or refute the use of these therapies.

Your help would be appreciated in the completion of this anonymous survey. Your involvement is voluntary and returning the completed questionnaire will indicate your consent to participate in this study. It should take about 20 minutes to complete the questionnaire. Please note that after submission, your survey cannot be withdrawn. The survey is confidential and the answers given by you are not identifiable.

When completing the survey please click on the box which best represents your answer. When you finish the survey, please click on the submit button. There are no right or wrong answers. The results will be published on the Polycystic Ovary Syndrome Association of Australia (POSAA) website.

This study is being carried out by a PhD student at the Complementary Medicine Research Centre at the University of Western Sydney. It has been approved by the Human Research Ethics Committee H 9431, of the University of Western Sydney.

If you have any questions regarding the survey please contact the researcher, Susan Arentz on 0403 044 247 or email [s.arentz@uws.edu.au](mailto:s.arentz@uws.edu.au). If you have any concerns or complaints about the ethical conduct or content of the survey, please contact the Office of Research Services on

Thank you for your participation.

What is Complementary Medicine? Complementary, alternative or natural medicine or therapies are those may include herbal tablets or capsules, vitamins and/or mineral supplements, food supplements such as fish oil, lecithin, spirilina or barley greens or visiting a complementary, traditional medicine or alternative practitioner. In this survey, complementary medicine or therapies is referred to as CM.

# To start we would like to ask you some questions about your general health and how you experience PCOS.

1. In general would you say that your physical health is (choose one only)

- Excellent
- Very good
- Good
- Fair
- Poor

1. Do you experience any of the following signs or symptoms that may be associated with PCOS?

- A late menstrual period
- Menstrual cramps
- Skin breakouts or skin irritation on the face, back or chest
- Depressed as a result of having PCOS
- Growth of visible hair on chin or body
- Waist larger than hips
- Overweight
- Very overweight
- Metabolic syndrome or insulin and glucose (or sugar) imbalances
- Infertility or taking longer than 12 months to get pregnant
- Other (please specify)...............................................................................................
- No signs or symptoms

1. During the past two weeks, how much of the time have you felt worried about having PCOS (choose as many as relate to your symptoms)

- All of the time
- Most of the time
- A good bit of the time
- Some of the time
- A little of the time
- Hardly any time
- Not any of the time

1. Do you keep a record of your menstrual period?

- Yes - always
- Yes -sometimes
- No

1. Have you tried any of the following to reduce or manage PCOS symptoms (please tick as many as needed)

- Exercise /physical activity
- Dietary changes
- Complementary medicine or natural therapies (CM)
- Pharmaceuticals or drugs such as Metformin or Clomid (clomiphene citrate)
- The oral contraceptive pill
- Fertility drugs as part of an IVF cycle
- Surgery for PCOS such as laparoscopic ovarian drilling or bariatric (stomach) surgery
- Other (please specify)..............................................................................................

# Now we want to ask you in more detail about ways, if any, that you have tried yourself to keep well or to manage the symptoms of PCOS. First some questions about complementary or natural therapies followed by some questions about diet, or the food you eat and some questions about exercise and physical activity.

# As a reminder, complementary medicine or therapies may be known to you as complementary, alternative or natural therapies.

1. In the last 12 months, have you used any of the following complementary medicines or natural therapies (CM’s)?

(More than once per day; daily; 4-6 times per week; 1-3 times per week; fortnightly; monthly; every 2^nd^ month; 6 monthly; every 9 months; once per year)

- Vitamins not prescribed by a doctor
- Other natural medicines from fish, plants or animals
- Herbal teas
- Mineral supplements not prescribed by a doctor
- Food supplements such as spirilina, Barley greens etc
- Mineral supplements prescribed by a doctor
- Herbal medicines or liquids such as Vitex agnes castus (chaste tree), Dong quai, Echinacea etc
- Other herbal medicines
- Traditional Chinese herbal tablets or teas
- Indigenous or other traditional medicines such as Ayevedic medicine
- Vitamins prescribed by a doctor
- Acupuncture
- Reflexology
- Aromatherapy oils for therapeutic reasons
- Homoeopathic medicines
- Other...............................................................................................................................
- No complementary medicines or natural health supplements used

1. Do you currently take or use any of the following CM’s?

- Vitamins not prescribed by a doctor
- Other natural medicines from fish, plants or animals
- Herbal teas
- Mineral supplements not prescribed by a doctor
- Food supplements such as spirilina, Barley greens etc
- Mineral supplements prescribed by a doctor
- Herbal medicines or liquids such as Vitex agnes castus (chaste tree), Dong quai, Echinacea etc
- Other herbal medicines
- Traditional Chinese herbal tablets or teas
- Indigenous or other traditional medicines such as Ayevedic medicine
- Vitamins prescribed by a doctor
- Acupuncture
- Reflexology
- Aromatherapy oils for therapeutic reasons
- Homoeopathic medicines
- Other...............................................................................................................................
- Not using complementary medicines or natural health supplements

1. Have you visited any of the following complementary practitioners or therapists?

- Acupuncturist
- Chiropractor
- Naturopath/western herbalist
- Osteopath
- Traditional Chinese medicine practitioner
- Homoeopath
- Other traditional medicine practitioner
- Reflexologist
- Massage or Bowen therapist
- Aromatherapist
- Iridologist
- Kinesiologist
- Other natural therapist (please specify)..........................................................................
- No, I have not visited a complementary practitioner or therapist

## If you have not used, are not currently using and have not visited a complementary practitioner or therapist, please go to question 13.

1. What area of your health are you currently using CM’s or seeing a complementary medicine or therapist for

- PCOS
- Increase energy
- Period pain
- PMS
- Fertility
- Sleep
- Digestion
- Constipation
- Acne
- Prevent getting colds and flu
- Stay healthy
- Depression
- Anxiety
- Other (please specify).........................................................................................................

1. If you are using CM’s for PCOS, what or who most influenced your decision to use complementary therapies? (Please tick as many as needed)

- I self prescribed
- Internet
- Retail assistant
- Family or friends
- Medical doctor
- Complementary medicine practitioner such as a naturopath, herbalist or acupuncturist
- Health professional such as a nurse, chemist, psychologist, osteopath, physiotherapist or other health provider
- Vitamin company hotline
- Magazines or books
- TV
- Other (please specify).........................................................................

1. Please tick if you have found CM’s effective for (check as many as required)

- Reducing PCOS symptoms such as regulating menstruation, or reducing acne
- Improving PCOS, in combination with diet and exercise
- Improving PCOS, in combination with drugs
- Reducing period pain
- Reducing PMS
- Improving general health
- Improving fertility
- Improving sleep
- Enhancing weight loss
- Other (please specify)..................................................................................................................
- No not effective at all
- Have not tried, so not sure

1. What do you think are the advantages of CM’s?

- More natural
- Lack of side effects
- Safe
- Effective, it seems to work
- Lasting effects
- Holistic effects
- Not addictive
- Can complement existing health care
- Can’t do harm
- I can be responsible for my own health and well-being
- I have not tried, so not sure
- Other......................................................................................................................................

1. What do you think are the main disadvantages of using CM’s?

- I don’t think there are any
- Expensive
- Takes longer to work
- Lack of research on if it works
- Lack of research on safety such as no warnings
- Not sure if it will work
- Not enough information
- Taste or smell of preparations
- Not confident to use in conjunction with medical drugs
- Not prescribed or recommended by my doctor
- Don’t know
- Other please specify...............................................

1. Have you ever had a negative reaction or unwanted side effects, to any complementary therapies?

- Yes
- No (please go to question 17)

1. If yes which complementary therapies?

- Vitamins not prescribed by a doctor
- Other natural medicines from fish, plants or animals
- Herbal teas
- Mineral supplements not prescribed by a doctor
- Food supplements such as spirilina, Barley greens etc
- Mineral supplements prescribed by a doctor
- Herbal medicines or liquids such as Vitex agnes castus (chaste tree), Dong quai, Echinacea etc
- Other herbal medicines
- Traditional Chinese herbal tablets or teas
- Indigenous or other traditional medicines such as Ayevedic medicine
- Vitamins prescribed by a doctor
- Acupuncture
- Reflexology
- Aromatherapy oils for therapeutic reasons
- Homoeopathic medicines
- Other...............................................................................................................................
- No complementary medicines or natural health supplements used

1. If yes, what has been the nature of the negative or unwanted side effects you experienced

(Temporary and I continued to take the CM; temporary symptoms went after I stopped the CM; symptoms continued after I stopped the herbal medicine);

- Menstrual cycle lengthened
- Menstrual cycle changed in a way I didn’t want
- Changes in bowel habits either looser or constipated
- Headaches
- Nausea
- Vomitting
- Skin rashes
- Sleep changes
- Heart beating fast
- Excessive sweating
- An anaphylactic reaction

# Next, we want to know whether or not you have tried any diets to improve the symptoms of PCOS or to help with weight loss or general well being.

1. In the past 5 years have you used any diets?

- Yes
- Maybe, I eat a healthy diet but not for any particular reason (please go to question 20)
- No (please go to question 22)

1. What was the purpose or reason for using the diet? (tick as many as appropriate)

- To lose weight
- To improve body composition (eg build muscle mass)
- To improve health
- To address a health concern (such as high blood sugar or high cholesterol)
- For social reasons, that is to enjoy with family or friends for example to encourage a family member to eat a particular diet
- Other (please specify)....................................................................................

1. Have you tried any of the following diets?

- Low Glycaemic Index (low GI) as in foods that lower blood sugar or glucose and lower insulin
- Low calorie
- Low Fat
- Energy restricting diet
- High protein
- Low carbohydrate
- CSIRO
- Liver detox diet
- Palm Beach diet
- Blood group diet
- Anti-candida diet
- Lean Cuisine
- Lite and Easy
- Protein powders
- Meal replacements
- Others (please specify) ..........................................................................................................
- No (please go to question 22)

1. If you were eating a special diet for a particular reason, do you feel that you achieved your goals?

- Yes
- No
- Don’t know

1. Have you experienced weight loss or weight management from any of the following diets?

- Low Glycaemic Index (low GI) as in foods that lower blood sugar or glucose and lower insulin
- Low calorie
- High protein
- Low carbohydrate
- Mediterranean
- Vegetarian
- CSIRO
- Liver detox diet
- Palm Beach Diet
- Blood group diet
- Anti-candida diet
- Lean Cuisine
- Lite and Easy
- Protein powders
- Meal replacement
- Other............................................................................................................

# Next are questions about exercise and physical activity, both moderate and vigorous activity. Moderate activity is movement that causes a slight increase in your breathing and heart rate, but you should still be able to talk. A brisk walk, mowing the lawn, or vacuuming are examples of moderate activity.

1. How many days per week do you

(once per week; twice per week three times per week four times per week; five or six times per week; everyday; more than once per day)

- Undertake moderate activity (such as walking)?
- Undertake vigorous activity such as aerobics, circuit training and running?

1. For how long do you usually undertake moderate physical activity?

- About 15 minutes
- About 30 minutes
- About an hour
- More than an hour

1. For how long do you undertake vigorous physical activity?

- About 15 minutes
- About 30 minutes
- About an hour
- More than an hour

1. Which forms of physical activity do you currently participate?

- Lift weights
- Brisk Walking up hills or stairs (where your breathing is heavy, panting and puffing)
- Run
- Group fitness classes at gym eg spin, zumba, pump
- Swim
- Personal trainer
- Dance
- Cycle
- Team sport such as netball, hockey etc
- Tennis (including table tennis)
- Incidental exercise (as part of a busy lifestyle- for example walking up stairs instead of taking the lift, parking the car a long way from your destination, gardening etc)
- Other................................................

1. Why do you undertake these forms of physical activity

- To feel better in my self
- To develop strength
- To lose weight
- To prevent weight gain
- Because I was advised to exercise by a health professional
- Because my family or friends suggested I did
- To meet specific goals or targets
- To get out of the house
- To socialise
- To set an example for my kids, family and/or friends
- To manage PCOS
- Other....................................................................................................

1. Do you feel that you have achieved your health goals with your exercise and physical activity?

- Yes
- Partly
- No
- Don’t know
- Comments...........................................................................

1. If physical activity is an ongoing part of your lifestyle, do you expect to be exercising this time next year?

- Yes
- No
- Don’ know

1. If you do not undertake moderate or vigorous activity, is there a particular reason?

- Limited time
- Not done before
- Physical Injury
- Feel self conscious or embarrassed
- No difference to my health
- No difference to my weight
- Increased my weight
- Trying to get pregnant
- I am pregnant
- Too difficult
- Not convinced of the benefits
- Limited money
- Other.............................................................................................................

# Finally some questions about you, to help us describe the women who have taken part in the survey.

1. Are you aged between

- 15 – 19
- 20-24
- 25-29
- 30-34
- 35-40
- 40-44
- 45+

1. Did you finish high school

- Yes
- No
- Still at high school

1. Have you completed any tertiary education

- Yes
- No

1. If yes please indicate if the qualification was

- A qualification from TAFE or similar
- University degree

1. In which country were you born

- Australia
- Other please specify...........................................................................................

1. Which of the following best describes your working activities (tick as many as required)

- Home duties
- Self employed
- Student
- Employed
- Other............................................................................................................................................

1. Your occupation takes

- 1 day per week
- 2-3 days per week
- 4-5 days per week
- 6-7 days per week

1. Do you have private health insurance?

- Yes
- No

Thank you very much for your help. Please remember to click on the submit button at the bottom of this page.
