# Supplementary material for: Perceptions and experiences of lifestyle interventions in women with polycystic ovary syndrome (PCOS), as a management strategy for symptoms of PCOS
Source: BMC Womens Health. 2021 Mar 17;21:107. doi: 10.1186/s12905-021-01252-1 (PMC7968330; doi:10.1186/s12905-021-01252-1)
Supplement: Supplementary file 2 — Additional file 2: Table 1: Comparison of early and and late responders and Table 2: Characteristics of POSAA and Facebook samples. [file 12905_2021_1252_MOESM2_ESM.docx]

Supplementary Tables 4 and 5.

Table 4. Comparison between early and late respondents in the POSAA group.

|  | Early respondents (b) (n=182) | | Late respondents (a)  (n=53) | | Difference % | |
| --- | --- | --- | --- | --- | --- | --- |
| Age | **%** | **95% CI** | **%** | **95% CI** | **a – b %** | **P value** |
| <17 years | 0.7 | ± 0.79 | 2.2 | ± 3.95 | 1.5 | 0.086 |
| 20-24 | 17.0 | ± 3.58 | 17.8 | ± 10.3 | -0.8 |  |
| 25-29 | 29.7 | ± 4.35 | 22.2 | ± 11.9 | -7.5 |  |
| 30-34 | 26.2 | ± 4.19 | 40.0 | ± 13.19 | 13.8 |  |
| 35-40 | 16.7 | ± 3.55 | 8.9 | ± 7.66 | -7.8 |  |
| 41-44 | 5.9 | ± 2.24 | 6.7 | ± 6.71 | -0.8 |  |
| >45 | 3.8 | ± 1.82 | 2.2 | ± 3.95 | -1.6 |  |
|  |  |  |  |  |  | |
| Education |  |  |  |  |  | |
| Finished high school | 83.3 | ± 5.42 | 80.6 | ± 10.65 | -4.2 | 0.497 |
| Tertiary education | 71.0 | ± 6.59 | 71.4 | ± 12.17 | -4.8 | 0.492 |
|  |  |  |  |  |  | |
| PCOS signs and symptoms all or most of the time | | | | | | |
| Late menstrual period | 69.7 | ± 6.68 | 68.5 | ± 12.51 | -1.2 | 0.815 |
| Hirsutism | 67.8 | ± 6.79 | 69.8 | ±12.36 | 2 | 0.782 |
| Overweight | 72.7 | ± 6.47 | 74.5 | ± 11.73 | 1.8 | 0.674 |
| Depression | 47.1 | ± 7.25 | 45.5 | ±13.41 | -1.6 | 0.842 |
|  |  |  |  |  |  | |
| Prevalence of use |  |  |  |  |  |  |
| Complementary medicine | 69.8 | ± 6.67 | 67.4 | ± 12.62 | -2.4 | 0.206 |

Table 5. Demographic and clinical characteristics of partcipants from two samples.

|  | POSAA sample  (n=182) | | Facebook sample  (n=311) | | P value |
| --- | --- | --- | --- | --- | --- |
|  | **%** | **95% CI** | **%** | **95% CI** |  |
| Demographics |  |  |  |  |  |
| Age 25-29 years | 31.6 | ± 6.77 | 27.9 | ± 4.98 | 0.588 |
| Finished high school | 83.3 | ± 5.42 | 87.9 | ± 3.62 | 0.165 |
| Employment full time | 53.1 | ± 7.25 | 39.8 | ± 5.44 | 0.543 |
|  | | | | | |
| Symptoms associated with PCOS all or most of the time | | | | | |
| Late menstrual period | 69.7 | ± 6.68 | 61.1 | ± 5.42 | 0.334 |
| Hirsutism | 67.8 | ± 6.79 | 66.2 | ± 5.26 | 0.880 |
| Overweight all or most of the time | 72.7 | ± 6.47 | 80.4 | ± 4.41 | 0.617 |
| Depression all or most of the time | 47.1 | 7.25 | 45.2 | ± 5.53 | 0.947 |
|  |  |  |  |  |  |
| Prevalence of use |  |  |  |  |  |
| Complementary medicine | 69.8 | ± 6.67 | 70.6 | ± 5.06 | 0.910 |
